# No friends 1

**DOI:** 10.1186/s13023-020-1327-7

**Published:** 2020-02-28

**Authors:** Anna-Lucia Koerling

**Affiliations:** University of Cambridge, Cambridge, UK

**Keywords:** Student, Essay, Competition, Neurofibromatosis 1, NF1, Depression, Mental health, Loneliness

## Abstract

Winning entry to the Student Voice 2019. The article focuses on a personal encounter that I had as a medical student when I was sent off to ‘study’ a patient with a rare disease who had been admitted to hospital.

**‘No Friends 1’**

Patients with rare diseases, particularly those with ‘useful’ signs for medical students, are often a hot commodity when inpatients in hospital. Supervisors dispatch us with a bed number and instructions to report our findings back to them, so we trot dutifully to far-flung wards with our stethoscopes slung around our necks, mentally reciting our spiel as we go;

‘Hi my name is Annie and I’m one of the fifth-year medical students … ’

David was one such patient. I was told to examine his peripheral nervous system, and as I headed towards his room I wondered what might be wrong with him. His date of birth, scribbled in my notebook along with his name and hospital number, suggested he was only 23. Cerebral Palsy? Muscular dystrophy? It was week two of my neurology rotation, and I’d mostly examined elderly people with strokes thus far, so I wasn’t sure what to expect.

David looked up and sighed ruefully as I entered his side room, putting his book down and giving me an amused smile.

‘Let me guess – a fifth year medical student on your neurology rotation? And you’d like to examine me?’

Thrown by this role reversal, I stared at him mutely, and nodded.

‘Annie’ I managed.

‘That’s fine, although just to let you know you’re the fourth one this morning, and it’s only 10:30’

I apologised, and made to leave, but he grinned and beckoned me inside, so I drew the curtain and nervously washed my hands, glancing surreptitiously at him. He was very thin, with dark brown hair cropped brutally short. His skin was so white as to be almost translucent - except that it was scattered with large pigmented areas the colour of tea with too much milk. They were everywhere, emerging from the sleeves of his hospital gown, climbing up the side of his neck, and bisecting his forehead. I tried not to stare, so looked around the sparse room, noticing his side table piled high with books, and a walking frame in the corner.

David was a model patient for examining, as he knew the examination routine better than I did. I found that he had leg weakness on both sides, with brisk reflexes and no sensation below his umbilicus. He also had some weakness and sensory loss in his left arm, which suggested a C6 radiculopathy (a compression of the nerve exiting the spinal cord). In combination with the pigmented areas of skin (usually known as café-au-lait macules), I was fairly sure he had neurofibromatosis type 1, and that his neurological symptoms were being caused by nerve sheath tumours or meningiomas in his spine.

Neurofibromatosis type 1 (NF1) is a syndrome caused by a mutation in the NF1 gene. One of many related conditions known as RASopathies, it has a has a panoply of possible manifestations, from the eponymous neurofibromas, to rarer symptoms such as cataracts and arterial stenosis [1]. Neurological development can be impaired, resulting in intellectual disability, and it increases the likelihood of multiple cancers. Life expectancy is not greatly reduced, but quality of life is lower in both children and adults than control groups, particularly with more morbid manifestations such as dystrophic scoliosis. It affects 1/3000 people, making it one of the commonest genetic syndromes in the world.

David confirmed that he had NF1 (‘or as I call it, No Friends 1’), including multiple spinal tumours, and congratulated me slightly sardonically on my deductions. I sat down to take a history, and he looked confused.

‘Ah. You’re the first one who’s wanted a chat’

I was surprised, and saddened. How awful it must have been to be David, constantly prodded and gawked at by medical students (most of whom were around his age), but unable to actually have a conversation with any of them. David was an expert patient, but I quickly realised that I’d prefer to hear about how NF1 had affected him than a disease summary I could learn from google.

‘So, how are you doing? Mentally, rather than physically?’

I watched as David’s eyes filled with tears, which he wiped embarrassedly on the blanket. It became apparent that in the 8 days since his admission, no one had asked him how he was dealing with the mental burden of his condition – something which is all too common for rare disease patients. In a survey conducted by Rare Disease UK [2], 46% of patients, and 58% of their carers report never being asked about their mental health/wellbeing. This is despite the well-established impact that living with a rare disease has on patients’ mental health. A meta-analysis of studies on the quality of life (QoL) of rare disease patients from 2010 showed that of the 58 studies included, a majority found that rare diseases patients had poorer quality of life [3]. Fourteen studies found that psychosocial quality of life was diminished in comparison with healthy controls, even if physical quality of life was not. The mental burden of living with a chronic illness should not be underestimated.

Furthermore, poor mental health is a comorbid factor in patients with chronic health conditions, worsening outcomes and quality of life [4]. Studies have shown that this effect is often worse in children and young people affected with a chronic condition – they feel more acutely the loss of their current (or future) functioning than older adults [5]. As most rare diseases affect young people [6], it seems evident that this is a population in need of more mental health support than others. However, this need goes largely unmet. Whilst patients are well supported physically by multidisciplinary teams, these often lack a psychiatric or psychological input, particularly in the UK, where mental health provisions are underfunded and inadequate in many areas [7].

Patients with NF1 are no exception. Multiple studies have shown poorer QoL in NF1 patients and carers than unaffected controls [8, 9], and psychosocial measures of QoL are particularly affected [8]. David had been on antidepressants since he was 17, and had had panic attacks since he was 7, usually triggered by anxiety about future manifestations of his condition. Now that he was bedbound, he told me he thought about suicide nearly every day, but didn’t feel like he could talk to anyone about it, particularly his parents. He was part of an NF1 forum on Facebook, but had stopped looking at it, as it made him angry to see people his age with NF1 who weren’t as profoundly disabled as he was. As he put it:

‘ This tumour is shredding my nerves day by day, both literally and figuratively’

David’s distress and frustration are shared by many rare disease patients. Studies have shown that loneliness [10], lack of social support [11], disempowerment, uncertainty about the future [12], and feelings of blame or loss [13] are all associated with feelings of depression and anxiety in rare disease patients.

Clinicians are capable of both exacerbating and ameliorating patients’ psychological distress, in all settings from GP to inpatient care. A patient’s GP making an effort to learn about a condition rather than relying on the patient for information alleviates somewhat the burden of responsibility that many patients feel for their own healthcare provision [2]. Similarly, specialists remembering to enquire about patients’ psychosocial well-being as well as their physical symptoms helps patients feel heard. Whilst individual changes in practice do little to eliminate the systemic change that needs to take place around provision of mental health services, they can make an enormous difference to patients.

Secondly, as was recommended by Rare Diseases UK in their report on mental health and rare diseases, healthcare professionals should signpost patients to sources of support. Patient support groups and charities exist for many rare diseases, and there are broader groups available for patients without a specific charity for their condition. Whilst these charities cannot always provide professional mental health support, talking to other patients or carers facing similar challenges can help reduce loneliness and uncertainty.

The biggest change, however, must occur at a national level. Both main parties have pledged to increase mental health funding should they win the 2019 general election. Whilst this is a welcome and necessary change, for rare disease patients it is not enough. The way we approach rare diseases must change, so that mental health assessment and treatment is commonplace, and a standard part of rare disease management. This will be difficult to implement, and requires coordinated engagement from every medical speciality, as well as from the NHS at an organisational level.

Talking to David taught me a lot about the kind of doctor I aspire to be. It can be easy to conflate a person with a pathology, but in doing so we lose sight of a fundamental truth – that our patients are first and foremost human beings. They crave conversation and human connection, just like the rest of us. I hope that my fellow future doctors and I will always remember to put ourselves in our patients’ shoes, and to ask a simple question:

‘How are you doing today?’

## Data Availability

No relevant data or materials.

## Abbreviations

NF1: Neurofibromatosis 1
QoL: Quality of life
